# Examining the Role of Teachers’ Stroking Behaviors in EFL Learners’ Active/Passive Motivation and Teacher Success

**DOI:** 10.3389/fpsyg.2021.707314

**Published:** 2021-07-20

**Authors:** Reza Pishghadam, Ali Derakhshan, Haniyeh Jajarmi, Sahar Tabatabaee Farani, Shaghayegh Shayesteh

**Affiliations:** ^1^Department of English, Ferdowsi University of Mashhad, Mashhad, Iran; ^2^Department of English Language and Literature, Faculty of Humanities and Social Sciences, Golestan University, Gorgan, Iran; ^3^Bahar Institute of Higher Education, Mashhad, Iran

**Keywords:** English as a foreign language, active motivation, passive motivation, teacher stroke, teacher success

## Abstract

Due to the important role that teachers’ professional success plays in the effectiveness of their students and the education system in which they are involved, the present study investigated whether teacher stroke can predict teacher success through the mediation of students’ active and passive motivation. For this aim, a group of 437 Iranian university English as a Foreign Language (EFL) students were targeted to respond to the teacher success, teacher stroke, and student motivation questionnaires. The main results of the study, obtained through running correlation and structural equation modeling (SEM), were first, while positive stroke showed a positive correlation with teacher success, it did not directly predict success; yet mediated by active motivation, it was a positive predictor of success; second, while teacher success had no significant relationship with total motivation, it was positively correlated with active and passive motivation, separately; third, in terms of gender differences, for the female participants, stroke, mediated by active motivation, was a better predictor of teacher success; fourth, high scores in positive, verbal, and conditional stroke were in association with high scores in active motivation, which significantly predicted teacher success. Based on the results, it can be concluded that teacher stroke, as an instance of positive teacher interpersonal communication behaviors, increases students’ active motivation for foreign language learning, which in turn results in their higher perceptions of English teachers’ professional success.

## Introduction

It is commonly believed that the teachers are the most important actors on the education scene (Pishghadam et al., 2021), and their professional success determines to a large degree the ultimate success of both students and the education system as a whole (Mercer and Dörnyei, 2020). This argument is well-captured in the title of Coombe’s (2020) recent article on the qualities of successful TESOL teachers, entitled as “Quality Education Begins with Teachers: What Are the Qualities That Make a TESOL Teacher Great?” Teacher success is in effect more prominent in the English as a Foreign Language (EFL) context where students’ opportunity to receive the target language input is largely limited to the confines of the classroom as English is not normally spoken in the Iranian community for daily and routine interactions (Pishghadam et al., 2019a). Within EFL classrooms, teachers play a key part, providing input to students, monitoring students’ progress, coordinating communication among students, providing feedback regarding students’ language production, and assessing students’ short- and long-term English language achievement. How successfully English language teachers engage in such undertakings depends on their level of professional effectiveness (Coombe, 2014).

Researchers in both domains of general education and language education have recognized the significance of teacher success since the early 1920s and have attempted to define, conceptualize, and put forward various frameworks and models on the characteristics of successful teachers (e.g., Demmon-Berger, 1986; Stronge, 2007; Elizabeth et al., 2008). It is argued that understanding what students perceive of their teachers’ success and exploring the teacher- or student-related factors contributing to this understanding are worthy areas of inquiry (Soodmand Afshar and Doosti, 2013). One of the student factors hypothesized to be influencing students’ perceptions of teacher success is learners’ level of perceived motivation. More particularly pertained to the context of the present study, which is the Iranian EFL context, Pishghadam et al. (2019b) have recently conceptualized English learning motivation to be comprised of two main elements of learner involvement and engagement, rooted in the four prominent theories of behaviorism, cognitivism, humanism, and social constructivism. A large body of research evidence strongly evinces that positive teacher verbal and non-verbal communication behaviors in the classroom greatly influence learners’ perceptions of their own level of motivation and their teachers’ professional performance (Frymier et al., 2019).

One of such positive teacher interpersonal communication variables is the newly introduced concept of teacher stroke. Rooted in the Transactional Analysis (TA) Theory of Eric Berne, teacher stroke is conceptualized as any action taken by the teacher to display his/her understanding of students’ presence and importance and to quench students’ always-present hunger for recognition on the part of the teacher (Shirai, 2006; Pishghadam and Farkhondehfal, 2017), which can be actualized through verbal, non-verbal, positive, or negative stroking cues (Pishghadam et al., 2019a). More specifically, perceived teacher stroke was found to be positively influencing a wide range of teacher- or student-related variables such as students’ perceptions of teacher credibility, teacher success, students’ willingness to attend classes, motivation, and foreign language learning (Pishghadam and Khajavy, 2014; Pishghadam and Karami, 2017; Rajabnejad et al., 2017; Pishghadam et al., 2021). Based on what was presented so far, thus, it would be plausible to hypothesize that first, there are associations among the three positive educational factors of teacher success, teacher stroke, and learner motivation, and second, teacher stroke, which is an instance of teacher positive communication behaviors toward students, can predict perceived teacher success through the mediation of learner motivation in the Iranian EFL context. The present study is conducted with the aim of empirically testing these research hypotheses.

## Literature Review

### Teacher Stroke

Positive relationship between the teacher and students may be closely connected with the emotions (especially the positive emotions highlighted in Positive Psychology) that students may experience during the process of second language acquisition (SLA) within language classes (Frenzel et al., 2009; McIntyre and Mercer, 2014; Dewaele et al., 2019; McIntyre et al., 2019). These emotions can bring about several changes, such as better students’ learning, enhanced motivation, and enriched interpersonal skills (Pierson, 2003; Peng and Woodrow, 2010; Khajavy, 2012). The bilateral teacher-student relationship can be evaluated through TA theory that was first introduced by Berne (1988) and was defined as a systematic theory of personal growth (Stewart and Joines, 1987). It can examine the interpersonal teacher-student relationships and enable students and the teacher to have productive communication resulting in a more attractive educational process (Stewart and Joines, 1987; Stuart and Alger, 2011; Barrow and Newton, 2015; Pishghadam et al., 2019a).

It is noteworthy to mention that the TA approach consists of six components: ego states, life positions, life scenario, transactions, time structures, and strokes (Berne, 1988). Stroke, one of the components of TA (Pishghadam et al., 2015, 2019a), is defined as indicating the awareness of the presence and values of others (Shirai, 2006), and the requirement for being admitted and noticed by others (Berne, 1988). Stroke is divided into verbal (ranging from a single word to a long conversation)/ non-verbal (activities like nodding or smiling), positive (using expressions such as *I love you*)/ negative (using expressions such as *I don’t want to see you again*), and conditional (what people do, e.g., *I enjoyed the music you played; you are not a good cook*)/ unconditional types (what people are, e.g., *I love you; I hate you*) (Stewart and Joines, 1987; Irajzad et al., 2017; Pishghadam et al., 2020).

Although it seems that stroking behaviors can be important in language classes, there is still a dearth of evidence on it. However, there have been some studies that could shed some light on its role in language classes (e.g., Pishghadam and Khajavy, 2014; Yazdanpour, 2015; Irajzad et al., 2017; Rajabnejad et al., 2017; Derakhshan et al., 2019; Pishghadam et al., 2019a). For instance, Pishghadam et al. (2019a) investigated the role of teacher success, credibility, and stroke in students’ willingness to attend classes (WTAC). The required scales were conducted, and the results of the analyses revealed that students’ WTAC could be significantly predicted by teacher success, credibility, and stroke. As another case in point, Pishghadam and Khajavy (2014) designed and validated the measure of student stroke to study the relationship between stroke and motivation, finding a positive correlation between the two.

### Active/Passive Motivation

Delving into the concept of motivation, specifically in the realm of second or foreign language (L2 or FL) teaching/learning, guides us to different theories rooted in Behaviorism, Cognitivism, Humanism, and Social Constructivism (Pishghadam et al., 2019b). Motivation is viewed as being connected with human being’s behavior and the external rewards (Behaviorism) (Staddon, 2001; Pishghadam et al., 2019b), the internal drives to do things (Humanism) (Maslow, 1943), the intrinsic/extrinsic classification based on the attractiveness of the results (Cognitivism) (Vroom, 1964; Porter and Lawler, 1968), and the social processes and people’s collective habits (Social Constructivism) (Bourdieu, 1986; Arnold and Walker, 2008; McCaslin, 2009; Pishghadam et al., 2019b). In addition, considering the significance of L2 motivation and taking a social-psychological approach to it, Gardner and Lambert (1972) introduced *integrative* (the tendency to become similar to the L2 group) and *instrumental* (the desire to achieve potential gains by the use of L2 proficiency) motivations (Dörnyei, 1994; Dörnyei and Csizér, 1998).

**FIGURE 1:**
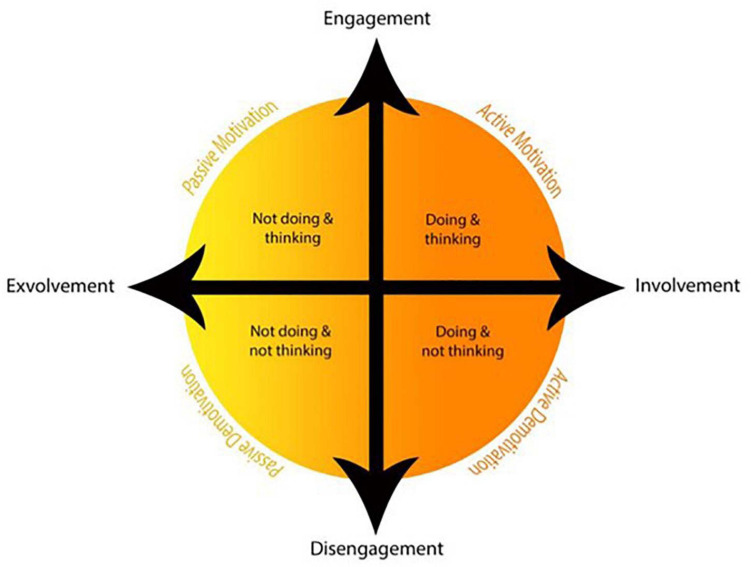
The Dual Continuum Model of Motivation. Adapted from “Unveiling the passive aspect of motivation: insights from English language teachers’ habitus,” by Pishghadam et al. (2019b), Copyright 2019 by the IJSCL.

Adopting a different perspective, Pishghadam et al. (2019b) introduced *the dual continuum model of motivation*; the model consists of engagement and involvement as the two fundamental constructs. Engagement is linked to the physical, cognitive, and emotional participation of individuals while performing a task (Kahn, 1990). Involvement is rooted in *emotioncy*, which refers to the emotions evoked by the senses employed in perceiving something; involvement is directly experiencing something or doing research on it to get more information (Pishghadam et al., 2016). Engagement is related to different degrees of sensory involvement that split “the model into two halves (i.e., active and passive) and four slices (comprising active motivation, active demotivation, passive motivation, and passive demotivation)” (Pishghadam et al., 2019b, p. 19). To further explain this model, active motivation is getting engaged in performing something; when this performance changes to a mechanical one due to the lack of mental engagement, it turns out to be active demotivation; passive motivation deals with the constant thinking about something but not performing it; and finally, passive demotivation pertains to exhibiting no cognitive or physical activity regarding a task (Pishghadam et al., 2019b) (Figure 1).

There is a paucity of empirical evidence on the concept of active/passive motivation, especially in language education. However, Pishghadam et al. (2019b) scrutinized the dichotomy by interviewing a group of English language teachers about the four conditions displayed in the model. In conclusion, it was revealed that the teachers’ habitus makes up the reasons for their passivity. Besides, Alami (2020) probed the relationships among the Iranian EFL learners’ active/passive motivation, their language learning motivation and demotivation, self-identity changes, and foreign language achievement. The analyses displayed the following results: first, significant relationships were found between active motivation and foreign language achievement and also between language learning motivation and foreign language achievement, and second, self-identity and language learning motivation were found to predict foreign language achievement.

### Teacher Success

Given that teachers are the main pillars of any educational system (Coombe, 2020), teacher success has gained remarkable notice in the realm of pedagogical research in which foreign language learning/teaching is not an exception. Teacher success can be defined as “the sense of achievement which teachers obtain from their work” (Hung et al., 2007, p. 415). More explicitly, the achievements may include getting a promotion, acquiring skills and knowledge, and improving patterns of behavior regarding relationships with students and instructional techniques, to name a few. Additionally, teachers’ attitudes, concerns, and expectations have been found to be related to their success (Hung et al., 2007).

To assess teacher success rigorously, some studies have tried to identify the constructs of teacher success via developing and validating different questionnaires (e.g., Khojastehmehr and Takrimi, 2009; Moafian and Pishghadam, 2009). Besides, another line of research focuses on the role of different variables in effective teacher performance. Essentially, research regarding the influential factors in relation to teacher success has set out the minimal explanatory adequacy of traditional benchmarks, such as certifications for identifying effective teacher performance (Duckworth et al., 2009). Hence, many studies have steered their focus toward factors such as language proficiency and pedagogical knowledge to identify personal traits pertinent to teacher success. In this regard, Derakhshan et al. (2020b) showed that EFL teachers’ positive attitudes toward research and their attention to the need for continuing professional development are two influential factors for teacher success. In another study, Derakhshan et al. (2020a) indicated that teachers’ professional identity and autonomy significantly and positively predict teacher success. Besides, Pishghadam et al. (2012) found a significant relationship between teacher creativity and success. Moreover, Duckworth et al. (2009) explicated that three positive characteristics of grit, optimistic explanatory style, and life satisfaction predict teacher success in terms of the academic achievements of students.

Overall, since teaching is an elaborate and challenging task, gaining success cannot be guaranteed in all situations; as a result, it is important to identify the factors facilitating or hindering teacher success (Hung et al., 2007). The evidence reviewed here suggests that little is known about the interrelationships of teacher stroke, EFL learners’ active/passive motivation, and teacher success. Moreover, our study differs from the previous ones in at least three respects. First, it explores the role of EFL learners’ preferred stroking patterns in the learners’ active/passive motivation and teacher success with respect to the learners’ gender. Second, while much of the literature on motivation pays particular attention to the active aspect of this construct, this study takes into account the passive aspect of EFL learners’ motivation as well. Finally, this study sought to inspect the mediating role of active/passive motivation in the relationship of teachers’ stroking behaviors and teacher success regarding which the previous literature lacks clarity. Accordingly, the current study addresses the following questions:

1. Are there any significant relationships among teacher stroke, Iranian EFL learners’ active/passive motivation, and teacher success?
2. Concerning the mediating role of Iranian EFL learners’ active/passive motivation, is teacher stroke a significant predictor of teacher success?
3. Concerning the mediating role of Iranian EFL learners’ active/passive motivation, is teacher stroke a significant predictor of teacher success with respect to the EFL learners’ gender?

## Materials and Methods

### Participants

The participants comprised 437 EFL learners (272 females and 165 males) studying at different universities in Mashhad, a city in the northeast part of Iran. They were BA or BSc students in different majors. Their ages ranged from 18 to 39 years of age (*M* = 20.33, *SD* = 2.66), and their English proficiency level ranged from low intermediate to advanced. They were selected based on convenience sampling and their willingness to take part in the study. The participants were ensured about the confidentiality of their information [based on British Educational Research Association, 2018] and received course credits for participation in the study.

### Instruments

The data for the current study were collected using three scales which were presented in Persian (i.e., the mother tongue of the participants).

#### Learner Stroke Quotient Scale (LSQS)

To measure the participants’ stroke quotient (i.e., preferred stroking patterns), the LSQS (Sakhtkar Haddadi, 2017) was used. The scale, validated through structural equation modeling (SEM) and confirmatory factor analysis (CFA), consists of 30 items, each describing an instance of a stroke type, being verbal unconditional positive (VUP, 5 items), verbal unconditional negative (VUN, 5 items), verbal conditional positive (VCP, 5 items), verbal conditional negative (VCN, 4 items), nonverbal positive (NP, 5 items), and nonverbal negative (NN, 6 items), through a Likert-type scale, ranging from -10 to +10. On the scale, -10 indicates the most negative stroke while +10 shows the most positive stroke, and zero indicates the insignificant stroke. Sakhtkar Haddadi (2017) reported the reliability coefficient of the scale to be.89. Sample items include “*When the teacher calls me by my first name*” (VUP), “*My teacher does not know my name. For example: While calling the roll, the teacher marks present or absent for others since she knows them but when it comes to my name she looks for me in the class*” (VUN), “*My teacher tries to get me involved in class discussion. ‘What do you think of this?*”’ (VCP), “*My teacher corrects me very boldly and on the spot*” (VCN), “*My teacher winks at me in class*” (NP), and “*My teacher sneers at my mistake*” (NN).

#### Active/Passive Motivation Scale (APMS)

The APMS (Alami, 2020) was used to measure the participants’ active and passive motivation. This scale was validated through SEM showing six sub-constructs of cognitive active motivation (CA, 4 items), cognitive passive motivation (CP, 4 items), socio-cultural active motivation (SoA, 4 items), socio-cultural passive motivation (SoP, 4 items), sensory active motivation (SeA, 4 items), and sensory passive motivation (SeP, 4 items). This 24-item questionnaire is a 6-point Likert-type scale, ranging from *strongly agree* (6) to *strongly disagree* (1). Alami (2020) reported an internal consistency of.90 for the scale. Sample items include “*I enjoy reading various English texts”* (CA), “*I am interested in expanding my vocabulary knowledge in different disciplines*” (CP), “*I enjoy group work in class*” (SoA), “*I am interested in working in foreign companies*” (SoP), “*I enjoy listening to lectures in English*” (SeA), and “*I am interested in watching English movies in cinema*” (SeP).

#### Characteristics of Successful EFL Teachers Questionnaire (CSTQ)

Teacher success was measured employing the CSTQ (Moafian and Pishghadam, 2009), which was validated through CFA. With a 5-point Likert-type scale ranging from *strongly agree* (5) to *strongly disagree* (1), this questionnaire contains 47 items encompassing 12 subscales, namely, teaching accountability (Acc, 7 items), interpersonal relationships (Int, 7 items), attention to all (Att, 5 items), examination (Ex, 3 items), commitment (Com, 3 items), learning boosters (Lea, 6 items), creating a sense of competence (Comp, 4 items), teaching boosters (Tea, 4 items), physical and emotional acceptance (Acp, 2 items), empathy (Emp, 2 items), class attendance (Atn, 2 items), and dynamism (Dyn, 2 items). Moafian and Pishghadam (2009) reported the Cronbach’s alpha reliability estimate of.94 for the scale. Sample items include “*S/he is interested in the subject s/he is teaching*” (Acc), “*S/he respects different opinions*” (Int), “*S/he is fair in evaluating and grading*” (Ex), and “*S/he has the ability to motivate language learners to learn the language*” (Comp).

### Procedure

Prior to filling out the questionnaires through the Google forms, the participants filled out a consent form and were ensured about the confidentiality of their information and the voluntary nature of their participation in the study. To collect the required data, the participants were asked by their teachers to complete the three scales included in one form. It took the participants approximately 30 min to answer the questionnaires. In this study, the reliability coefficient of the scales was calculated through the Cronbach’s alpha procedure. Moreover, using AMOS (Version 24), SEM was used to estimate the possible predictability of teachers’ stroking behaviors through active/passive motivation and teacher success.

## Results

### Descriptive Statistics

Descriptive statistics for LSQS, APMS, and CSTQ can be seen in Table 1. Given that the Skewness and Kurtosis values were within the range of −2 and +2, the normal distribution of the data was confirmed. Reliability coefficients were further calculated.

**TABLE 1:** Descriptive Statistics and Reliability Estimates for LSQS, APMS, and CSTQ.

	Min	Max	M	SD	Skewness	Kurtosis	Reliability
**LSQS**							
Verbal Unconditional Positive	7.00	21.00	12.96	2.05	1.05	1.36	0.90
Verbal Unconditional Negative	1.00	21.00	6.78	4.03	1.01	0.81	0.88
Verbal Conditional Positive	3.80	21.00	12.38	2.27	0.20	1.47	0.79
Verbal Conditional Negative	1.00	21.00	9.14	3.98	0.32	–0.41	0.75
Nonverbal Positive	3.00	21.00	12.46	2.80	–0.05	0.73	0.81
Nonverbal Negative	1.83	21.00	9.22	3.35	0.63	0.51	0.82
**APMS**							
Cognitive/Active Motivation	4.00	24.00	18.17	4.03	–0.70	0.22	0.88
Cognitive/Passive Motivation	5.00	24.00	19.20	3.65	–0.89	0.85	0.86
Socio-Cultural/Active Motivation	7.00	24.00	19.80	3.87	–0.85	0.06	0.91
Socio-Cultural/Active Motivation	4.00	24.00	19.84	4.09	–1.15	1.17	0.79
Sensory/Active Motivation	4.00	24.00	19.84	3.78	–1.13	1.44	0.81
Sensory/Passive Motivation	4.00	24.00	19.12	4.65	–0.91	0.09	0.85
**CSTQ**							
Teaching Accountability	2.43	5.00	4.61	0.48	–1.61	1.70	0.91
Interpersonal Relationships	1.71	5.00	4.57	0.49	–1.83	1.50	0.92
Attention to All	1.80	5.00	4.53	0.60	–1.69	1.24	0.89
Examination	1.67	5.00	4.25	0.75	–.78	–.23	0.79
Commitment	2.67	5.00	4.59	0.50	–1.19	0.85	0.88
Learning Boosters	1.67	5.00	4.38	0.63	–1.19	1.42	0.83
Creating a Sense of Competence	1.00	5.00	4.03	0.75	–0.62	0.21	0.85
Teaching Boosters	2.00	5.00	4.46	0.52	–1.18	1.76	0.88
Physical and Emotional Acceptance	3.00	5.00	4.65	0.52	–1.28	0.47	0.76
Empathy	1.00	5.00	4.68	0.57	–1.45	1.81	0.78
Class Attendance	3.00	5.00	4.79	0.44	–1.30	1.76	0.76
Dynamism	1.50	5.00	4.42	0.68	–1.17	1.05	0.77

### Correlational Analysis

In order to find possible relationships among teacher success, teacher stroke, and active/passive motivation, the Pearson product-moment correlation was used. Based on Table 2, teacher success (*r* = 0.11, *p* < 0.05) and four of its sub-constructs, namely learning boosters (*r* = 0.12, *p* < 0.01), creating a sense of competence (*r* = 0.11, *p* < 0.05), empathy (*r* = 0.10, *p* < 0.05), and dynamism (*r* = 0.12, *p* < 0.01), have significant correlations with giving stroke. There is also positive relationships between teacher success and the negative sub-constructs of LSQS, namely VUN (*r* = 0.11, *p* < 0.05), VCN (*r* = 0.10, *p* < 0.05), and NN (*r* = 0.11, *p* < 0.05). While teacher success has no significant relationship with total motivation, it is positively correlated with active (*r* = 0.19, *p* < 0.01) and passive motivation (*r* = 0.10, *p* < 0.01), separately.

**TABLE 2:** Correlational Analyses for the Variables.

	1	2	3	4	5	6	7	8	9	10	11	12	13	14	15	16	17	18	19	20	21	22	23	24	25	26	27	28	29
**LSQS**	1																												
VUP	0.55**	1																											
VUN	0.85**	0.28**	1																										
VCP	0.68**	0.49**	0.43**	1																									
VCN	0.84**	0.28**	0.74**	0.50**	1																								
NP	0.64**	0.53**	0.34**	0.53**	0.39**	1																							
NN	0.83**	0.29**	0.76**	0.37**	0.72**	0.35**	1																						
**AMPS**	0.05	0.01	0.02	0.05	0.01	0.07	0.04	1																					
Active	–0.00	0.01	–0.00	–0.01	–0.05	0.05	–0.01	0.11*	1																				
Passive	–0.02	–0.02	–0.02	–0.03	–0.04	0.03	–0.03	0.10*	0.78**	1																			
CA	0.012	–0.00	–0.00	–0.00	–0.03	0.05	0.00	0.12**	0.89**	0.64**	1																		
CP	–0.04	–0.05	–0.05	–0.01	–0.07	0.05	–0.06	0.12*	0.60**	0.78**	0.55**	1																	
SoA	0.00	–0.01	0.00	–0.00	–0.03	0.04	–0.01	0.12**	0.85**	0.67**	0.65**	0.53**	1																
SoP	–0.01	–0.03	0.00	–0.03	–0.02	0.01	–0.02	0.07	0.68**	0.88**	0.55**	0.55**	0.62**	1															
SeA	–0.02	0.05	–0.02	–0.03	–0.08	0.03	–0.02	0.05	0.85**	0.72**	0.66**	0.49**	0.57**	0.61**	1														
SeP	–0.01	0.00	–0.01	–0.03	–0.03	0.01	–0.00	0.08	0.70**	0.88**	0.55**	0.52**	0.56**	0.70**	0.72**	1													
**CTSQ**	0.11*	0.01	0.11*	0.09	0.10*	0.02	0.11*	0.03	0.19**	0.10**	0.19**	0.20**	0.20**	0.14**	0.09*	0.17**	1												
Acc	0.09	0.03	0.08	0.09*	0.09*	0.01	0.08	0.00	0.12**	0.15**	0.13**	0.16**	0.14**	0.10*	0.05	0.13**	0.90**	1											
Int	0.10*	0.02	0.11*	0.10*	0.07	0.00	0.09*	0.02	0.08	0.07	0.09*	0.11*	0.10*	0.03	0.02	0.04	0.85**	0.77**	1										
Att	0.09	0.00	0.11*	0.04	0.09	0.01	0.08	0.00	0.11*	0.13**	0.12*	0.16**	0.13**	0.10*	0.04	0.08	0.85**	0.79**	0.67**	1									
Ex	0.07	–0.02	0.08	0.05	0.08	0.01	0.07	0.09*	0.23**	0.28**	0.21**	0.20**	0.23**	0.21**	0.16**	0.30**	0.75**	0.63**	0.53**	0.59**	1								
Com	0.02	–0.01	0.00	0.04	0.03	–0.01	0.04	0.01	0.07	0.11*	0.11*	0.14**	0.09	0.11*	–0.00	0.05	0.68**	0.63**	0.57**	0.55**	0.45**	1							
Lea	0.12**	0.02	0.13**	0.08	0.12**	0.01	0.12**	0.01	0.18**	0.17**	0.18**	0.19**	0.19**	0.10*	0.08	0.16**	0.92**	0.84**	0.71**	0.77**	0.68**	0.56**	1						
Comp	0.11*	0.03	0.11*	0.08	0.07	0.03	0.12**	0.06	0.28**	0.27**	0.29**	0.23**	0.26**	0.19**	0.18**	0.28**	0.81**	0.66**	0.63**	0.62**	0.65**	0.45**	0.75**	1					
Tea	0.06	0.01	0.03	0.06	0.04	0.01	0.07	0.05	0.23**	0.24**	0.22**	0.22**	0.24**	0.19**	0.14**	0.21**	0.83**	0.79**	0.68**	0.63**	0.59**	0.58**	0.73**	0.69**	1				
Acp	0.05	0.00	0.00	0.05	0.07	0.00	0.09*	0.08	0.13**	0.16**	0.10*	0.16**	0.16**	0.08	0.08	0.17**	0.69**	0.64**	0.55**	0.52**	0.56**	0.52**	0.57**	0.49**	0.59**	1			
Emp	0.10*	0.05	0.08	0.06	0.09*	0.06	0.08	–0.01	0.08	0.06	0.11*	0.15**	0.09	0.01	–0.00	0.01	0.67**	0.60**	0.60**	0.0.60**	0.48**	0.41**	0.58**	0.43**	0.51**	0.45**	1		
Atn	0.03	–0.06	0.01	0.04	0.04	0.03	0.03	–0.01	0.04	0.10*	0.06	0.18**	0.04	0.04	0.00	0.04	0.61**	0.59**	0.54**	0.56**	0.38**	0.46**	0.55**	0.33**	0.44**	0.53**	0.41**	1	
Dyn	0.12**	0.04	0.11*	0.08	0.11*	0.04	0.12**	0.00	0.15**	0.09	0.18**	0.09	0.15**	0.06	0.07	0.08	0.82**	0.75**	0.67**	0.67**	0.59**	0.53**	0.76**	0.70**	0.68**	0.56**	0.50**	0.43**	1

**. Correlation is significant at the 0.01 level (2-tailed).

*. Correlation is significant at the 0.05 level (2-tailed).

*VUP, Verbal Unconditional Positive; VUN, Verbal Unconditional Negative; VCP, Verbal Conditional Positive; VCN, Verbal Conditional Negative; CA, Cognitive Active; CP, Cognitive Passive; SoA, Socio-cultural Active; SoP, Socio-cultural Passive; SeA, Sensory Active; SeP, Sensory Passive; Acc, Teaching Accountability; Int, Interpersonal Relationships; Att, Attention to All; Ex, Examination; Com, Commitment; Lea, Learning Boosters; Comp, Creating a Sense of Competence; Tea, Teaching Boosters; Acp, Physical and Emotional Acceptance; Emp, Empathy; Atn, Class Attendance; Dyn, Dynamism).*

### SEM

Structural equation modeling (SEM) models were proposed to verify the predictive power of stroke through the mediation of active/passive motivation. Goodness of fit indices showed that the models fit the data adequately (see Table 3).

**TABLE 3:** Goodness of Fit Indices for the Models.

	χ^2^/df	df	CFI	TLI	RMSEA	SRMR
Model 1 (Figure 2)	4.47	222	0.91	0.91	0.07	0.08
Model 2 (Figure 3A)	3.15	231	0.91	0.90	0.07	0.08
Model 3 (Figure 3B)	2.56	234	0.90	0.90	0.08	0.08
Model 4 (Figure 4)	4.34	237	0.90	0.90	0.07	0.07
Model 5 (Figure 5A)	2.91	231	0.92	0.91	0.06	0.07
Model 6 (Figure 5B)	2.40	231	0.90	0.90	0.07	0.08
Model 7 (Figure 6)	4.46	224	0.91	0.90	0.07	0.07
Model 8 (Figure 7A)	3.10	224	0.92	0.91	0.07	0.07
Model 9 (Figure 7B)	2.74	224	0.90	0.90	0.08	0.8
Model 10 (Figure 8)	4.59	182	0.91	90	0.07	0.07
Model 11 (Figure 9A)	3.38	185	0.92	0.92	0.07	0.07
Model 12 (Figure 9B)	2.99	202	0.90	0.90	0.08	0.08

#### Stroke as a Whole

**FIGURE 2:**
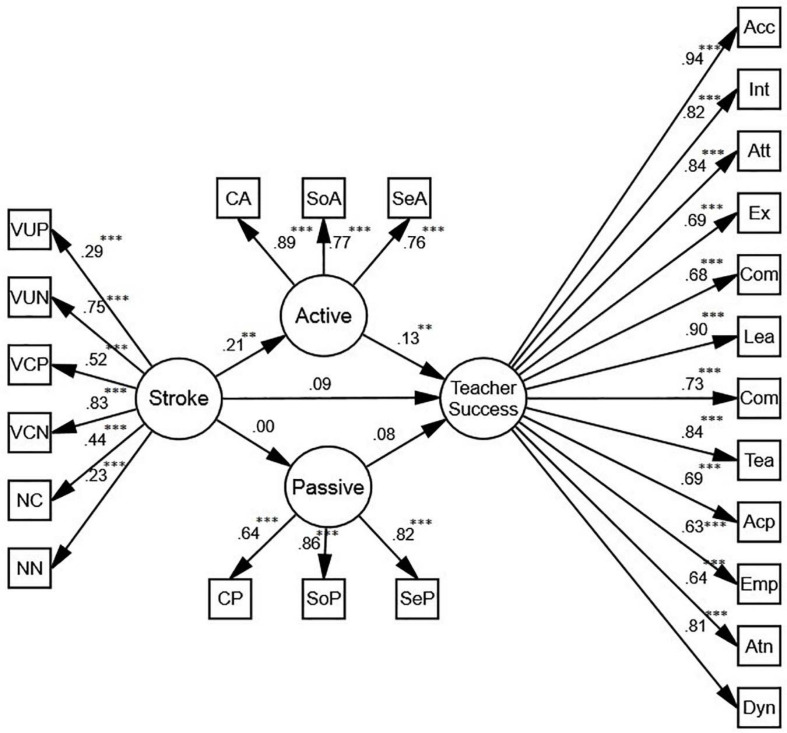
The schematic representation of the relationships among stroke, active and passive motivation, and teacher success (** *p* < 0.01, *** *p* < 0.001). In order to see if gender of the participants changes the reported relationships, two more SEM models were proposed.

The first model (Figure 2) shows that stroke does not predict teacher success directly (β = 0.09, *p* > 0.05, *R*^2^ = 0.03); yet when mediated by active motivation, it is a positive predictor of teacher success (β = 0.13, *p* < 0.01, *R*^2^ = 0.05).

**FIGURE 3:**
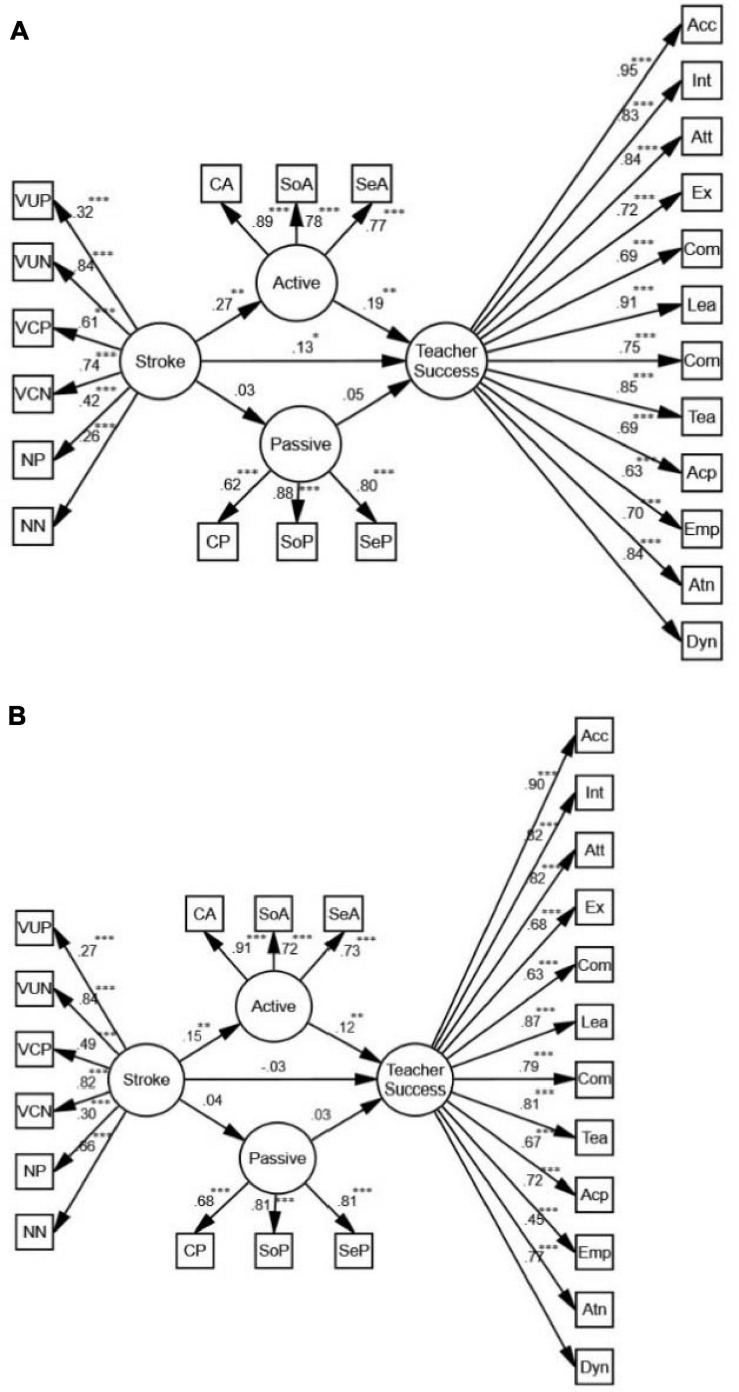
**(A)** The schematic representation of the relationships between stroke, active and passive motivation, and teacher success (Females). **(B)** The schematic representation of the relationships between stroke, active and passive motivation, and teacher success (Males) (** *p* < 0.01, *** *p* < 0.001).

Based on the model for females (Figure 3A), stroke is a positive predictor of teacher success both directly (β = 0.13, *p* < 0.05, *R*^2^ = 0.07) and indirectly (β = 0.19, *p* < 0.01, *R*^2^ = 0.07). Mediated by active motivation, the predictive power is stronger than the direct one. With regards to males (Figure 3B), however, stroke may only predict teacher success when mediated by active motivation (β = 0.12, *p* < 0.01, *R*^2^ = 0.05). Comparing the models for males and females, we conclude that for the female participants, stroke is a better predictor of teacher success. It should be noted that there exists no significant relationship between stroke, passive motivation, and teacher success for either males or females.

In order to be more specific and examine the relationships among different forms of stroke (including positive/negative, verbal/nonverbal, and conditional/unconditional), active and passive motivation, and teacher success, several SEM models were proposed. The role of gender was additionally taken into account.

#### Positive/Negative Stroke

**FIGURE 4:**
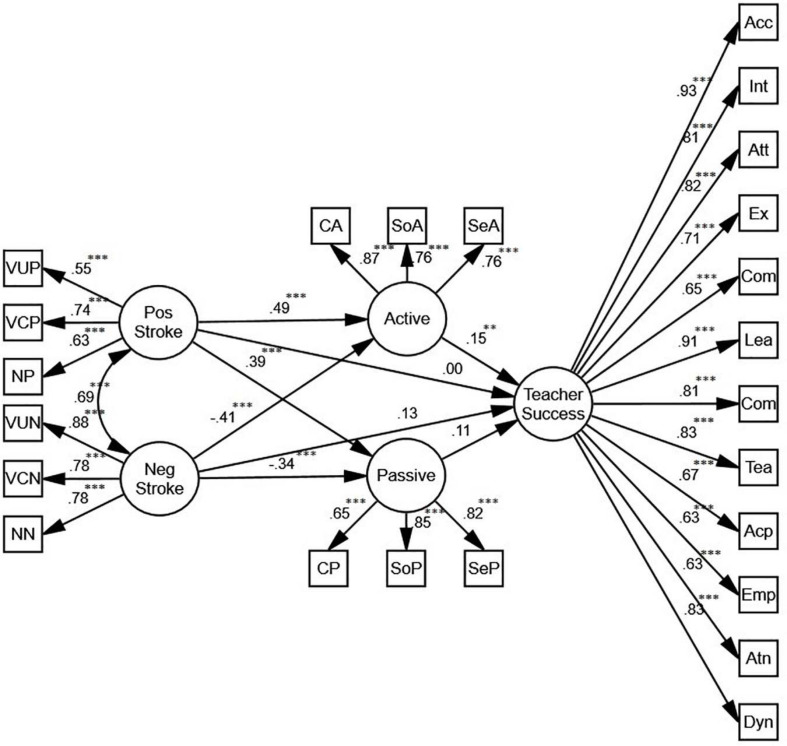
The schematic representation of the relationships between positive (pos) and negative (neg) stroke, active and passive motivation, and teacher success (** *p* < 0.01, *** *p* < 0.001).

Figure 4 shows that positive and negative stroke, mediated by active motivation, predict teacher success (β = 0.15, *p* < 0.01, *R*^2^ = 0.05). While the relationship between active motivation and positive stroke is positive (β = 0.49, *p* < 0.001, *R*^2^ = 0.13), the relationship between active motivation and negative stroke is negative (β = −0.41, *p* < 0.01, *R*^2^ = 0.13). There is also a positive correlation between passive motivation and positive stroke (β = 0.39, *p* < 0.001, *R*^2^ = 0.11), and a negative correlation between passive motivation and negative stroke (β = −0.34, *p* < 0.001, *R*^2^ = 0.11) which do not predict teacher success. Overall, while the relationships between positive stroke and active and passive motivation are positive, the relationships between negative stroke and active and passive motivation are negative. In order to verify the role of gender, two more SEM models were proposed.

**FIGURE 5:**
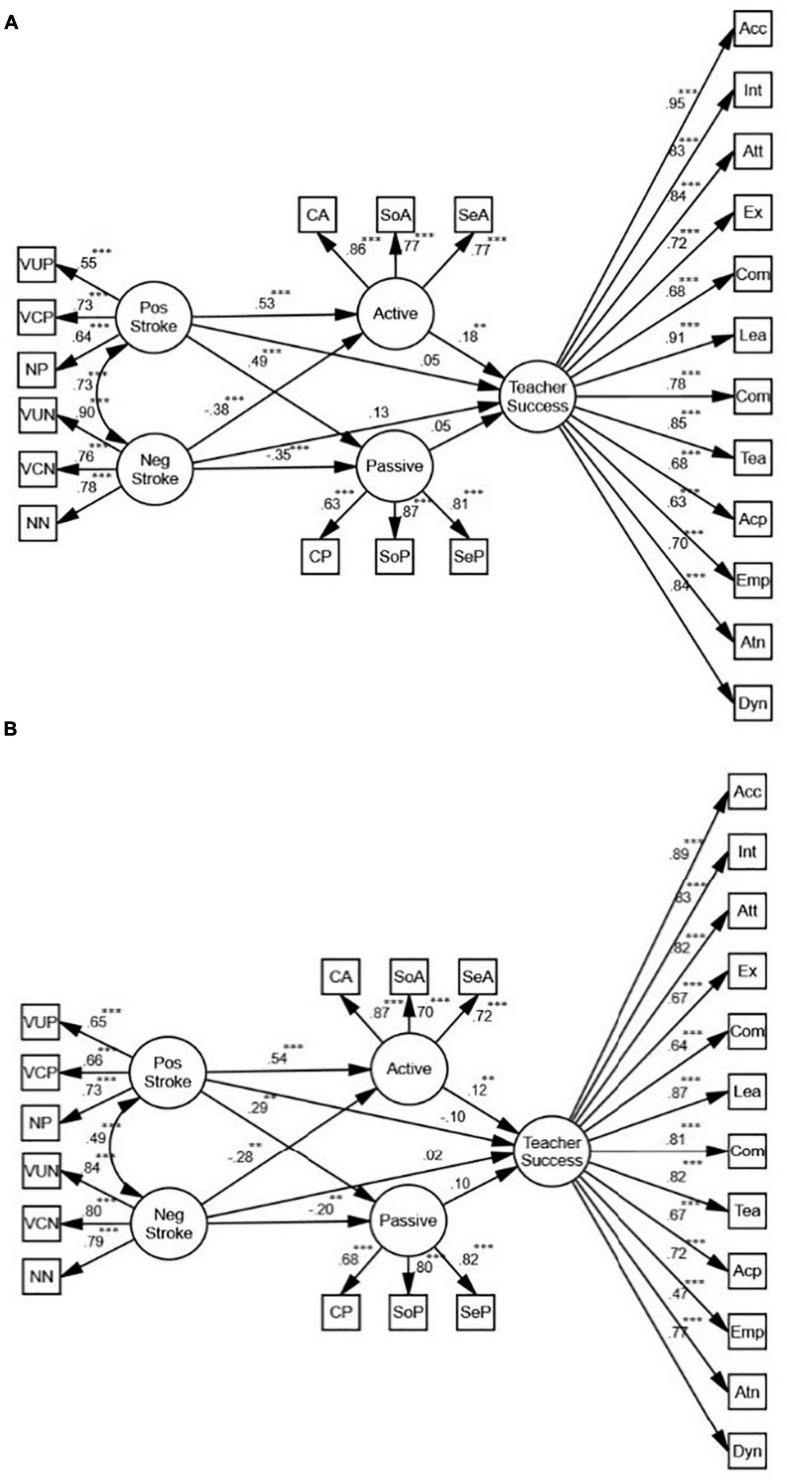
**(A)** The schematic representation of the relationships between positive (pos) and negative (neg) stroke, active and passive motivation, and teacher success (Females). **(B)** The schematic representation of the relationships between positive and negative stroke, active and passive motivation, and teacher success (Males) (** *p* < 0.01, *** *p* < 0.001).

Based on the models for females and males (Figures 5A,B), positive stroke is an indirect, positive predictor of teacher success. Mediated by active motivation, the predictive power for females (β = 0.18, *p* < 0.01, *R*^2^ = 0.07) is stronger than the one for males (β = 0.12, *p* < 0.01, *R*^2^ = 0.05). In both models, there is also a positive correlation between passive motivation and positive stroke (β = 0.49, *p* < 0.001, *R*^2^ = 0.05; β = 0.29, *p* < 0.01, *R*^2^ = 0.07), and a negative correlation between passive motivation and negative stroke (β = −0.35, *p* < 0.001, *R*^2^ = 0.05; β = −0.20, *p* < 0.01, *R*^2^ = 0.07) which do not predict teacher success.

#### Verbal/Nonverbal Stroke

**FIGURE 6:**
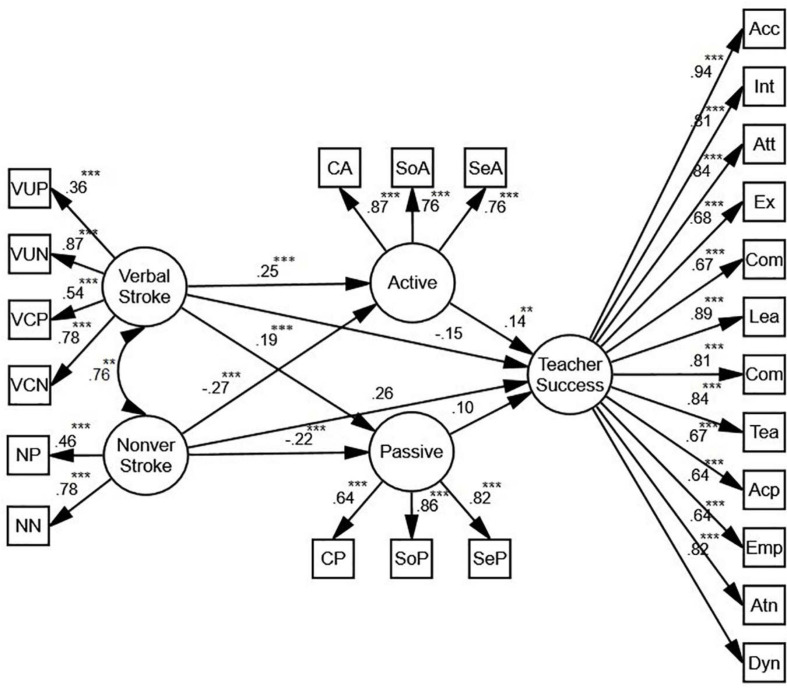
The schematic representation of the relationships between verbal and nonverbal (nonver) stroke, active and passive motivation, and teacher success (** *p* < 0.01, *** *p* < 0.001).

Figure 6 shows that verbal and nonverbal stroke, mediated by active motivation, predict teacher success (β = 0.14, *p* < 0.01, *R*^2^ = 0.05). While the relationship between active motivation and verbal stroke is positive (β = 0.25, *p* < 0.001, *R*^2^ = 0.05), the relationship between active motivation and nonverbal stroke is negative (β = −0.27, *p* < 0.001, *R*^2^ = 0.05). There is also a positive correlation between passive motivation and verbal stroke (β = 0.19, *p* < 0.001, *R*^2^ = 0.05), and a negative correlation between passive motivation and nonverbal stroke (β = −0.22, *p* < 0.001, *R*^2^ = 0.05) which do not predict teacher success. Overall, while the relationships between verbal stroke and active and passive motivation are positive, the relationships between nonverbal stroke and active and passive motivation are negative. In order to verify the role of gender, two more SEM models were proposed.

**FIGURE 7:**
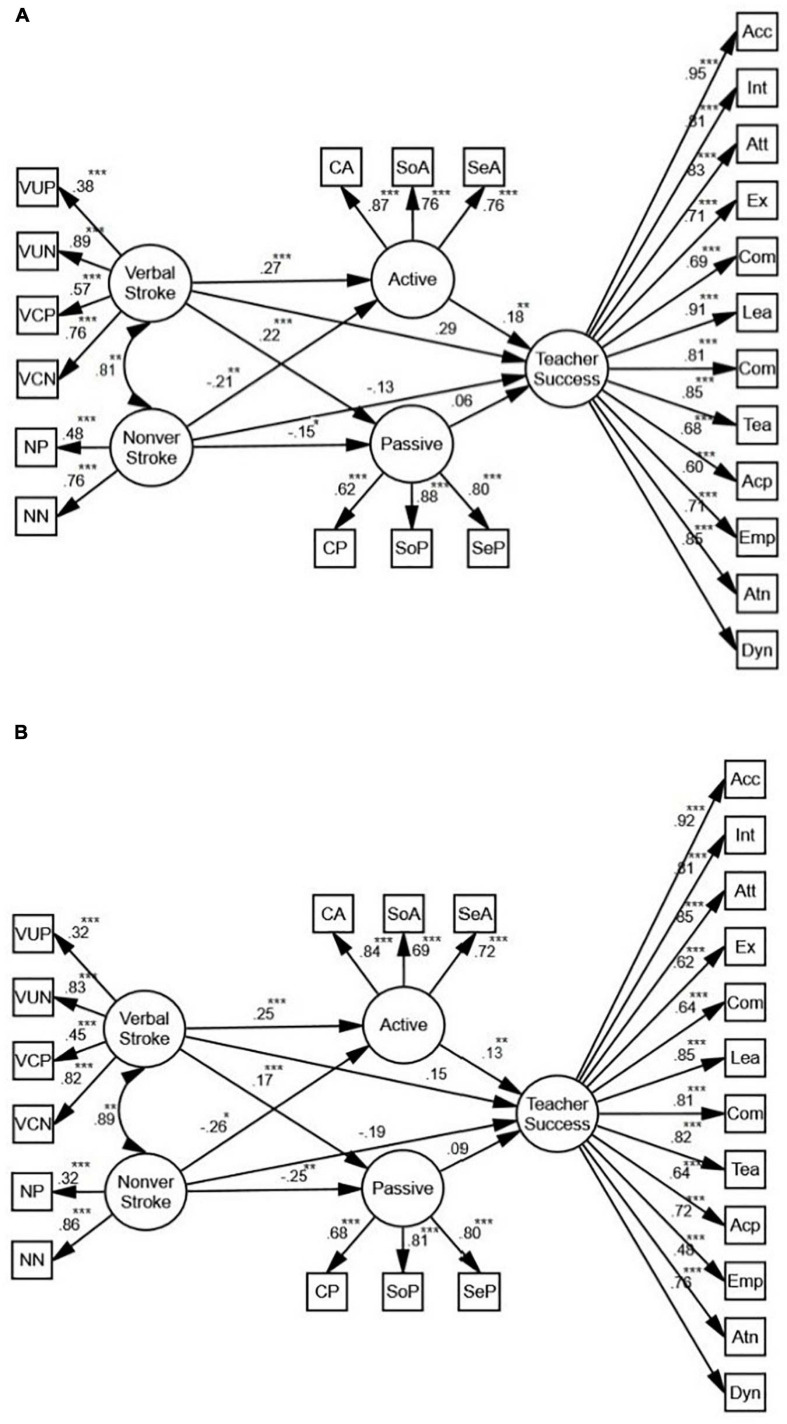
**(A)** The schematic representation of the relationships between verbal and nonverbal (nonver) stroke, active and passive motivation, and teacher success (Females). **(B)** The schematic representation of the relationships between verbal and nonverbal (nonver) stroke, active and passive motivation, and teacher success (Males) (* *p* < 0.05, ** *p* < 0.01, *** *p* < 0.001).

Based on the models for females and males (Figures 7A,B), verbal stroke is an indirect, positive predictor of teacher success. Mediated by active motivation, the predictive power for females (β = 0.18, *p* < 0.01, *R*^2^ = 0.06) is stronger than the one for males (β = 0.13, *p* < 0.01, *R*^2^ = 0.05). In both models, there is also a positive correlation between passive motivation and verbal stroke (β = 0.22, *p* < 0.001, *R*^2^ = 0.05; β = 0.17, *p* < 0.001, *R*^2^ = 0.05), and a negative correlation between passive motivation and nonverbal stroke (β = −0.15, *p* < 0.05, *R*^2^ = 0.05; β = −0.25, *p* < 0.01, *R*^2^ = 0.05) which do not predict teacher success.

#### Conditional/Unconditional Stroke

**FIGURE 8:**
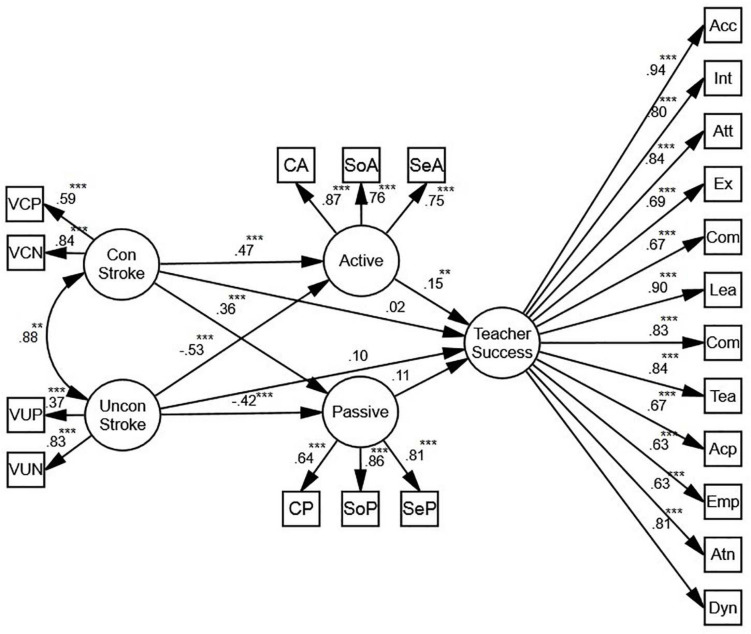
The schematic representation of the relationships between conditional (con) and unconditional (uncon) stroke, active and passive motivation, and teacher success (** *p* < 0.01, *** *p* < 0.001).

Figure 8 shows that, mediated by active motivation, conditional and unconditional stroke predict teacher success (β = 0.15, *p* < 0.01, *R*^2^ = 0.07). While the relationship between active motivation and conditional stroke is positive (β = 0.47, *p* < 0.001, *R*^2^ = 0.06), the relationship between active motivation and unconditional stroke is negative (β = −0.53, *p* < 0.001, *R*^2^ = 0.06). There is also a positive correlation between passive motivation and conditional stroke (β = 0.36, *p* < 0.001, *R*^2^ = 0.05), and a negative correlation between passive motivation and unconditional stroke (β = −0.42, *p* < 0.001, *R*^2^ = 0.05) which do not predict teacher success. Overall, while the relationships between conditional stroke and active and passive motivation are positive, the relationships between unconditional stroke and active and passive motivation are negative. In order to verify the role of gender, two more SEM models were proposed.

**FIGURE 9:**
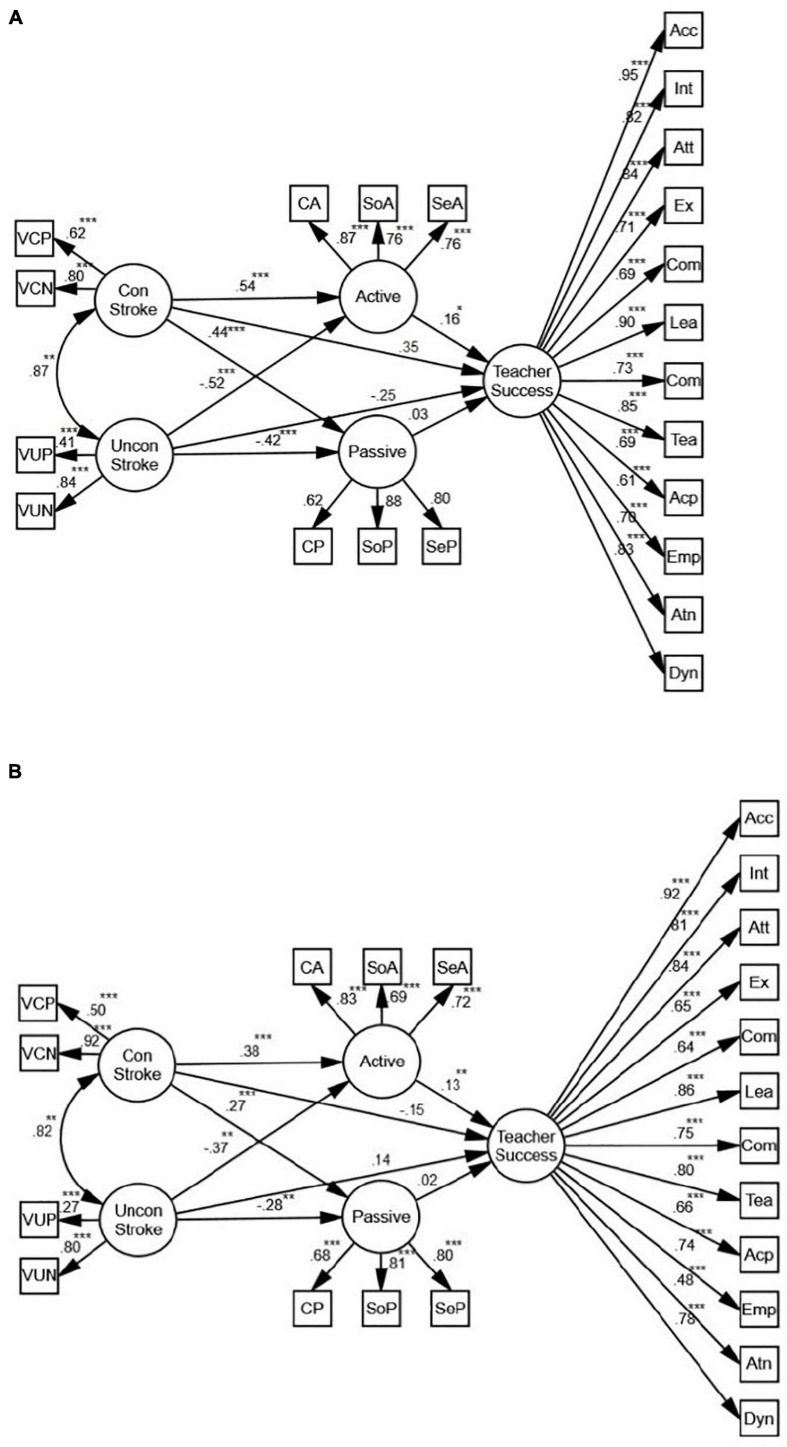
**(A)** The schematic representation of the relationships between conditional (con) and unconditional (uncon) stroke, active and passive motivation, and teacher success (Females). **(B)** The schematic representation of the relationships between conditional (con) and unconditional (uncon) stroke, active and passive motivation, and teacher success (Males) (* *p* < 0.05, ** *p* < 0.01, *** *p* < 0.001).

Based on the models for females and males (Figures 9A,B), conditional stroke is an indirect, positive predictor of teacher success. Mediated by active motivation, the predictive power for females (β = 0.16, *p* < 0.06, *R*^2^ = 0.11) is stronger than the one for males (β = 0.13, *p* < 0.01, *R*^2^ = 0.08). In both models, there is also a positive correlation between passive motivation and conditional stroke (β = 0.44, *p* < 0.001, *R*^2^ = 0.05; β = 0.27, *p* < 0.001, *R*^2^ = 0.06), and a negative correlation between passive motivation and unconditional stroke (β = −0.42, *p* < 0.001, *R*^2^ = 0.05; β = −0.28, *p* < 0.01, *R*^2^ = 0.06) which do not predict teacher success.

To see whether the models fit the data, goodness of fit indices were calculated using AMOS. Table 3 shows the relative chi-square [i.e., chi-square index divided by the degrees of freedom (χ^2^/df)], Comparative Fit Index (CFI), Tucker-Lewis Index (TLI), Root Mean Square Error of Approximation (RMSEA), and Standardized Root Mean Squared Error (SRMR). The criterion for acceptance is different across researchers. In the present study, values for χ^2^/ df were within the acceptable limit of 5 or less (Hair et al., 2010), TLI and CFI were over 0.90, and RMSEA and SRMR were equal to or less than 0.08 (Browne and Cudeck, 1993); thus, the models fit the data adequately.

## Discussion

This study aimed at investigating the role of Iranian EFL learners’ preferred stroking patterns in the learners’ active/passive motivation and teacher success. In this regard, the objectives of this study were, first, to examine the interrelationships among the three variables; second, to determine if teacher stroke, with the mediating role of learners’ active/passive motivation, is a significant predictor of teacher success; and finally, to identify the probable differences between male and female EFL learners regarding the predictability of teacher success by teacher stroke, taking into account the mediating role of active/passive motivation.

With respect to the first research question, we found that, for teachers, giving stroke had a significantly positive correlation with gaining success. This finding is consistent with those of other studies (Pishghadam and Karami, 2017; Noorbakhsh et al., 2018; Amini et al., 2019; Pishghadam et al., 2019a, 2021) where stroke positively correlated with teacher success. Moreover, it is in line with Hattie and Timperley (2007) assertion that providing verbal feedback, here as a type of stroke, is a positive indicator of successful teachers. More specifically, we found a significantly positive correlation among teacher stroke and the four teacher success components of learning boosters, creating a sense of competence, empathy, and dynamism. This is in agreement with the findings of other studies, in which the application of stroke, as an instance of positive teacher interpersonal communication behaviors, in educational contexts has been associated with positive changes in the learning process (Frymier and Houser, 2000; Stuart and Alger, 2011; Wubbels et al., 2016; Frymier et al., 2019; McIntyre et al., 2020), enhanced motivation and sense of competence (Pierson, 2003; Lawson and Lawson, 2013; Pishghadam and Khajavy, 2014), and teacher creativity, dynamism (Pishghadam et al., 2012), care toward students, and feedback (Derakhshan et al., 2019).

On the other hand, although motivation has been a prolific area of investigation, to date, the concept of active/passive motivation has not yet been closely studied and a systematic understanding of how EFL learners’ active/passive motivation contributes to teacher success is still lacking. Hence, a particularly remarkable finding of this study is related to the second research question, based on which the mediating role of active/passive motivation was investigated in the predictability of teacher success by stroke, in general, and by different forms of stroke (i.e., positive/negative, verbal/nonverbal, and conditional/unconditional), in particular.

Surprisingly, contrary to the findings of the previous studies (Pishghadam and Karami, 2017; Noorbakhsh et al., 2018; Amini et al., 2019; Pishghadam et al., 2021), in this study, stroke did not predict teacher success directly, yet its positive prediction of teacher success was mediated by active motivation. On close inspection, the results of the current study showed that if teacher stroke engenders active motivation, that is, get students involved in performing something rather than just thinking about it, then it will lead to teacher success. More explicitly, it seems that the stroke which invokes passive motivation will not lead to teacher success. This supports Stewart and Joines’s (1987) argument that teacher stroking behaviors reinforce the positive behavior and performance of the person provided with stroke. Moreover, this finding is partially in line with those of previous studies (Francis and Woodcock, 1996; Pierson, 2003; Lawson and Lawson, 2013; Pishghadam and Khajavy, 2014) in which teacher stroke was found to be in association with motivation; but the point is that these studies considered motivation as a unitary concept and overlooked the passive aspect of motivation.

Given the significance of cultural perceptions and experiences in the process of foreign language learning and teaching (Baumgratz-Gangl, 1990; Pishghadam et al., 2021), this intriguing finding could be attributed to the prevailing collectivist culture of the Iranians. As Asian countries tend to be cohesive regarding interpersonal relationships (Hofstede, 2007), thus, it seems that individuals may not receive adequate attention in these societies and may constantly feel ignored (Pishghadam et al., 2020). Accordingly, since collectivism is characterized by “‘We’-consciousness” and “languages in which the word ‘I’ is avoided” (Hofstede, 2011, p. 11), it can be inferred that teacher stroke creates a sense of being recognized which would be desirable to students in that their learning and academic engagement is perceived to be important to the teacher (McIntyre et al., 2020), which in turn leads to the generation of active motivation and boosts teacher success.

Additionally, to be more specific, enjoying SEM, we examined the relationships among different forms of stroke (including positive/negative, verbal/nonverbal, and conditional/unconditional), active/passive motivation, and teacher success. Regarding the positive/negative stroke, the results indicated that positive and negative stroke, mediated by active motivation, predicted teacher success. In other words, high scores in positive stroke were in association with high scores in active motivation and high scores in negative stroke were associated with low scores in active motivation, which significantly predicted teacher success. These results are likely to be related to the traces of emotionality in collectivist cultures such as the Iranian culture (Hofstede, 1980, 2007) in which people tend to be acknowledged positively by receiving positive stroke rather than receiving negative stroke. Hence, teacher positive stroke can potentially increase students’ active motivation, which in turn increases their perceptions of teacher success. This finding is in line with those of Amini et al. (2019) and Cohen and Steele’s (2002) studies, indicating that positive stroke is a significant predictor of teacher success; however, their findings differ from our findings as they did not take into account active motivation as the mediator variable in the relationship of teacher stroke with success.

With respect to the verbal/nonverbal stroke, we found that high scores in verbal stroke and in nonverbal stroke were in association with high scores and low scores in active motivation, respectively, which significantly predicted teacher success. Put it simply, the results showed that when verbal stroke increases active motivation, it consequently leads to higher perceptions of teacher success. These results could be interpreted in light of an outstanding cultural theory, distinguishing high-context cultures from low-context ones (Hall, 1976). Based on Hall’s context theory, ways of communication differ from one culture to another, ranging from explicit (low-context culture) to implicit (high-context culture). From this respect, the capability of comprehending messages depends on individuals’ cultural background (Hall and Hall, 1990). Essentially, due to the fact that a high-context culture prevails in Iran, non-verbal language (i.e., tone of voice, facial expression, eye contact, and gestures) carry notable meanings in conversations and verbal message is indirect and implicit, meaning that context conveys more meaning than words (Hall, 1976). It is, therefore, likely that giving verbal stroke in a prevailing high-context culture engenders active motivation in students which in turn, leads to higher perceptions of teacher success. This outcome is in line with that of Pishghadam and Karami’s (2017) study whichh revealed that from among the four forms of stroke, verbal stroke had the highest correlation with teacher success. However, it is worth mentioning that in the literature, no detailed investigation of the role of different forms of stroke, taking the mediating role of active motivation, in teacher success was found.

Regarding the conditional/unconditional dimensions of stroke, the current study found that high scores in conditional stroke and in unconditional stroke were in association with high scores and low scores in active motivation, respectively, which significantly predicted teacher success. That is, conditional stroke, in case of producing active motivation, will lead to teacher success. One possible explanation for this outcome is the collectivist culture of the Iranians. Since Asian cultures are basically relationship-based (Hofstede, 2007), providing students with unconditional stroke may result in the misunderstanding of this form of stroke in that it may seem not to be reasonable and to be based on some sort of rapport or teacher-student relationship. On the other hand, this outcome can be supported based on the principle of fairness stating that “personal and social circumstances –for example gender, socio-economic status or ethnic origin– should not be an obstacle to achieving educational potential” (Field et al., 2007, p. 11). Accordingly, unconditional stroke may seem not to be fair and to be based on, for example, the strokee’s gender or socio-economic status, which gives more credit to the conditional stroke. Another possible explanation for this finding is the psychological need of individuals for receiving stroke as the result of what they have done rather than receiving it unconditionally. The results of this study underpin this idea that the statement of reasons based on which students receive stroke leads to active motivation and eventually to increased perceptions of teacher success.

The results pertaining to the third research question showed that, compared with males, for the female participants, stroke, mediated by active motivation, was a better predictor of teacher success. More specifically, mediated by active motivation, the predictive power of positive, verbal, and conditional forms of stroke for females were stronger than the ones for males. This implies that compared with males, female students are more sensitive to teacher stroke. It is noteworthy to say that no research has been found that surveyed the strokability of males in comparison to females in educational contexts with regard to the resultant active motivation leading to teacher success. However, in line with the findings of this study, similar studies corroborate the need of individuals to be stroked (Pishghadam and Ghahari, 2012; Pishghadam et al., 2019a), and that stroking is a crucial component of teacher care (Pishghadam et al., 2015).

Overall, the strengths of this study included the in-depth quantitative analysis of the mediating role of active/passive motivation in the relationship of teacher stroke with success by focusing specifically on the sub-constructs of teacher stroke and their effects on the female and male EFL learners’ perceptions of successful teachers. Additionally, the present study adds to the growing body of research that indicates the significance of learners’ cultural backgrounds in shaping and reshaping their perceptions of effective teachers (Pishghadam et al., 2021). The evidence from this study recommends teachers to exert more care not only about the form of stroke they provide to their students but also about whom (male or female) they are giving stroke. On the other hand, it should be noted that the generalizability of these results is subject to certain limitations. For instance, the participants were recruited through convenience sampling from among the Iranian university students; therefore, further work is required to establish the viability of the proposed models. Moreover, we carried out this research considering the participants’ gender. Future studies on the current topic are, therefore, recommended to elucidate the interrelationships among the variables considering learners’ age and socio-economic status, among other demographic information variables.

## Data Availability Statement

The raw data supporting the conclusions of this article will be made available by the authors, without undue reservation.

## Ethics Statement

The studies involving human participants were reviewed and approved by the Ferdowsi University of Mashhad Ethics Committee. The patients/participants provided their written informed consent to participate in this study.

## Author Contributions

RP: conceived and designed the experiments. HJ, SS, and ST: performed the experiments. SS and HJ: analyzed the data. RP, HJ, and AD: contributed reagents, materials, and analysis tools. HJ, SS, ST, and AD: wrote ıthe manuscript. RP and AD: reviewed and edited. All authors contributed to the article and approved the submitted version.

## Conflict of Interest

The authors declare that the research was conducted in the absence of any commercial or financial relationships that could be construed as a potential conflict of interest.
